# Supplementary material for: Tectonically-triggered sediment and carbon export to the Hadal zone
Source: Nat Commun. 2018 Jan 9;9:121. doi: 10.1038/s41467-017-02504-1 (PMC5760703; doi:10.1038/s41467-017-02504-1)
Supplement: Supplementary file 1 — Supplementary Information [file 41467_2017_2504_MOESM1_ESM.pdf]

Supplementary Table 1. Bulk and RPO data (TOC content, radiocarbon, and stable carbon isotopic characteristics of bulk OC and thermochemical fractions) in selected samples (A – E) from core GeoB 16431-1.

No.	Core depth (cm)	TOC (wt %)	Bulk			T_1 (170-320°C)			T_2 (320-391°C)			T_3 (391-476°C)			T_4 (476-570°C)			T_5 (570-915°C)		
			$\delta^{13}C$	Fm	^{14}C age	$\delta^{13}C$	Fm	^{14}C age	$\delta^{13}C$	Fm	^{14}C age	$\delta^{13}C$	Fm	^{14}C age	$\delta^{13}C$	Fm	^{14}C age	$\delta^{13}C$	Fm	^{14}C age
A	5-7	1.7	-25.1	0.7943 ±0.0105	1850 ±107	-23.8	0.8392 ±0.0018	1410# ±15	-22.3	0.7747 ±0.0082	2050 ±85	-24.4	0.6856 ±0.0078	3032 ±91	-26.5	0.6874 ±0.0073	3011 ± 85	-28.3	0.5649 ±0.0065	4588 ±93
B	352-355	1.2	-28.7	0.5222 ±0.0095	5218 ±146	-25.3	0.5426 ±0.0058	4912 ±85	-24.7	0.5042 ±0.0054	5501 ±87	-28.7	0.3962 ±0.0048	7437 ±97	-29.4	0.4147 ±0.0049	7071 ±95	-30.1	0.3352 ±0.0044	8780 ±104
C	448-451	1.5	-26.2	0.6766 ±0.0101	3139 ±120	-24.0	0.7032 ±0.0017	2830# ±20	-23.2	0.6646 ±0.0087	3282 ±105	-21.9	0.5600 ±0.0057	4658 ±81	-27.7	0.6091 ±0.0067	3983 ±88	-33.2	0.4953 ± 0.0060	5644 ±98
D	544-548	0.6	-30.9	0.3093 ±0.0088	9425 ±228	-25.6	0.3132 ±0.0043	9326 ±111	-26.7	0.2656 ±0.0037	10648 ±113	-28.0	0.2200 ±0.0040	12164 ±145	-36.5	0.2319 ±0.0037	11740 ±127	-33.2	0.1850 ±0.0044	13553 ±193
E	615-618	1.6	-27.5	0.6691 ±0.0102	3228 ±122	-23.4	0.6929 ±0.0020	2950# ±25	-21.9	0.6357 ±0.0063	3639 ±79	-25.2	0.5481 ±0.0056	4830 ± 82	-25.6	0.5270 ±0.0057	5145 ±86	-25.2	0.4876 ±0.0059	5770 ±97

Note: # indicates that samples were measured at NOSAMS.

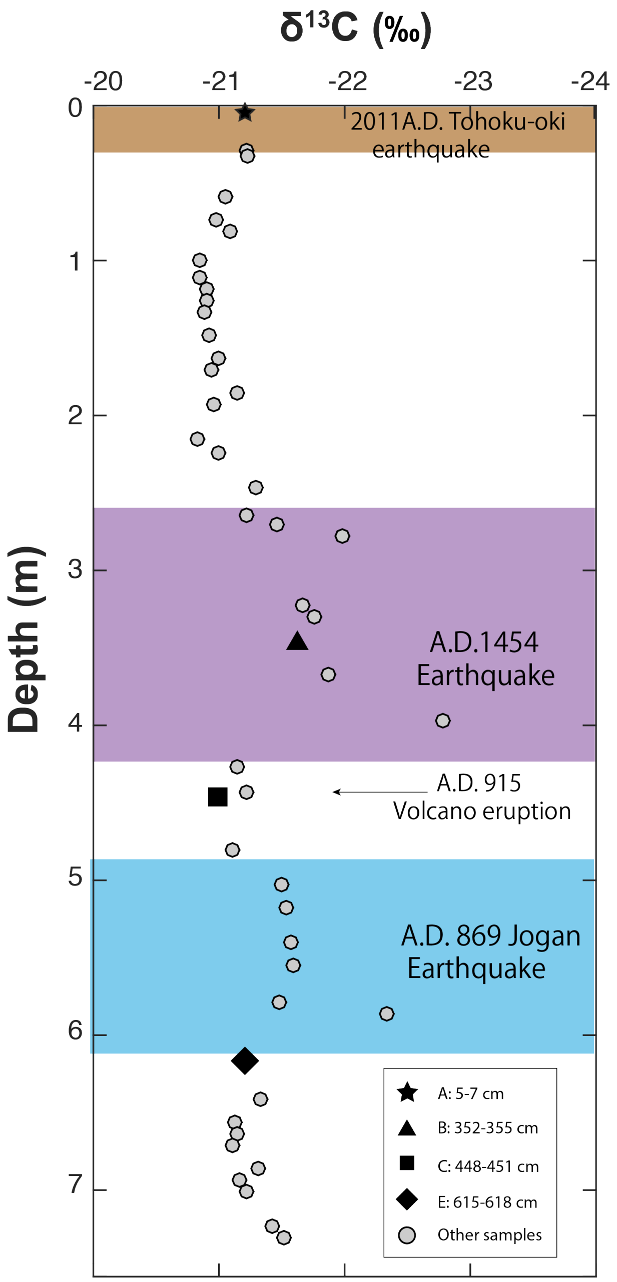

Supplementary Figure 1. Bulk OC $\delta^{13}C$ profile of Core GeoB 16431-1(n = 47). The $\delta^{13}C$ of sample D was not gained due to its measurement failure. The samples for OC ^{13}C analysis in Ag capsules were first fumigated (37%HCl for 72 hrs) and measured with a ± 0.2 ‰ precision using a coupled elemental analyzer (EA) / IRMS / AMS online system at ETH Zürich (McIntyre et al., 2016).

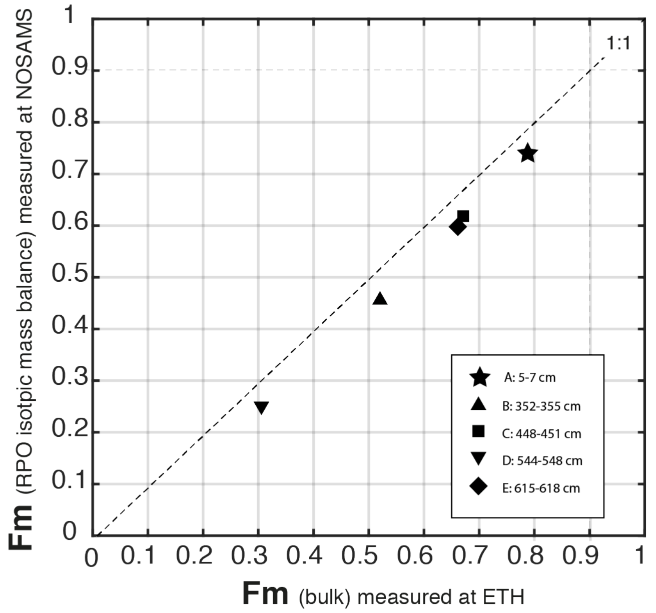

Supplementary Figure 2. Isotopic relationship between calculated (from RPO isotopic mass balance) and measured bulk values for Fm values at NOSAMS and ETH, respectively.

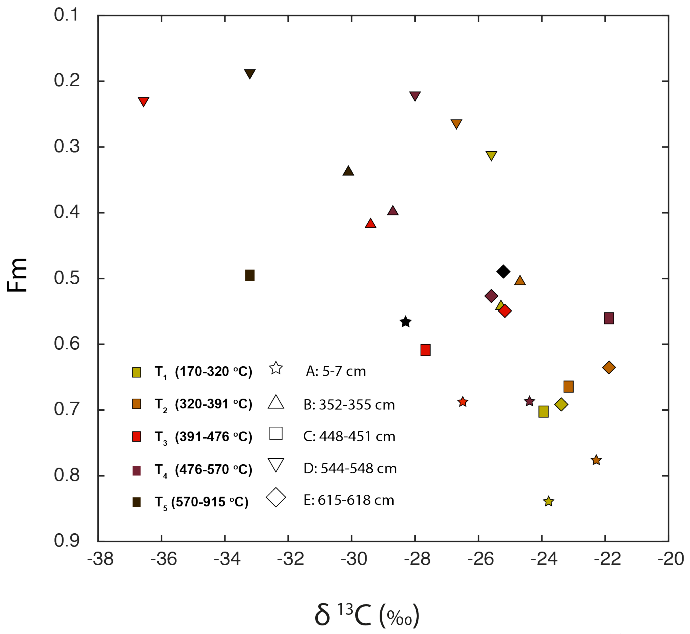

Supplementary Figure 3. Cross-plot between Fm values and $\delta^{13}C$ data of RPO fractions (T_1-T_5) among the five samples.

Supplementary reference:

1. McIntyre, C. P. et al. Online ^{13}C and ^{14}C gas measurements by EA–IRMS–AMS at ETH Zürich. *Radiocarbon* **58,** 1–7 (2016).
