# Supplementary material for: Tectonically-triggered sediment and carbon export to the Hadal zone
Source: Nat Commun. 2018 Jan 9;9:121. doi: 10.1038/s41467-017-02504-1 (PMC5760703; doi:10.1038/s41467-017-02504-1)
Supplement: Supplementary file 3 — Description of Additional Supplementary Files [file 41467_2017_2504_MOESM3_ESM.pdf]

Description of Additional Supplementary Files

File Name: Supplementary Data 1
Description: High-resolutions data (bulk radiocarbon, TOC content, density, and calculated sedimentation rate and OC fluxes) from core GeoB 16431-1.
